# Chondrosarcoma-from Molecular Pathology to Novel Therapies

**DOI:** 10.3390/cancers13102390

**Published:** 2021-05-14

**Authors:** Agnieszka E. Zając, Sylwia Kopeć, Bartłomiej Szostakowski, Mateusz J. Spałek, Michał Fiedorowicz, Elżbieta Bylina, Paulina Filipowicz, Anna Szumera-Ciećkiewicz, Andrzej Tysarowski, Anna M. Czarnecka, Piotr Rutkowski

**Affiliations:** 1 Department of Soft Tissue/Bone Sarcoma and Melanoma, Maria Sklodowska-Curie National Research Institute of Oncology, 02-781 Warsaw, Poland; agnieszka.zajac@pib-nio.pl (A.E.Z.); sylwia.kopec@pib-nio.pl (S.K.); bartlomiej.szostakowski@pib-nio.pl (B.S.); mateusz.spalek@pib-nio.pl (M.J.S.); elzbieta.bylina@pib-nio.pl (E.B.); paulinafilipowicz7@gmail.com (P.F.); piotr.rutkowski@pib-nio.pl (P.R.); 2 Small Animal Magnetic Resonance Imaging Laboratory, Mossakowski Medical Research Institute, Polish Academy of Sciences, 02-106 Warsaw, Poland; mfiedorowicz@imdik.pan.pl; 3 Department of Clinical Trials, Maria Sklodowska-Curie National Research Institute of Oncology, 02-781 Warsaw, Poland; 4 Faculty of Medicine, Medical University of Warsaw, 02-091 Warsaw, Poland; 5 Department of Pathology and Laboratory Diagnostics, Maria Sklodowska-Curie National Research Institute of Oncology, 02-781 Warsaw, Poland; anna.szumera-cieckiewicz@pib-nio.pl; 6 Department of Diagnostic Hematology, Institute of Hematology and Transfusion Medicine, 02-776 Warsaw, Poland; 7 Department of Pathology and Laboratory Medicine, Maria Sklodowska-Curie National Research Institute of Oncology, 02-781 Warsaw, Poland; andrzej.tysarowski@pib-nio.pl; 8 Department of Molecular and Translational Oncology, Maria Sklodowska-Curie National Research Institute of Oncology, 02-781 Warsaw, Poland

**Keywords:** chondrosarcoma, diagnostic markers, pathology, therapies

## Abstract

**Simple Summary:**

Chondrosarcoma (CHS) belongs to a broad group of sarcomas and is the second most frequent malignant bone tumor. Due to its resistance to chemo- and radiotherapy, treatment of this tumor is complicated and is mainly limited to surgery. In this review, we described the characteristics of CHS comprehensively from its molecular basics, through diagnosis, and finally to treatment methods with emphasizing the novel potential therapies and currently ongoing clinical trials. We discussed the potential of targeted therapies, including blockers of crucial in pathogenesis receptors, mutated genes inhibitors, and capabilities of immunotherapy. Ultimately, in this review we summarized the present possibilities in CHS treatment and its outcomes with novel trends which can become a point of interest in future researches on CHS.

**Abstract:**

Chondrosarcoma (CHS) is the second most common primary malignant bone sarcoma. Overall survival and prognosis of this tumor are various and often extreme, depending on histological grade and tumor subtype. CHS treatment is difficult, and surgery remains still the gold standard due to the resistance of this tumor to other therapeutic options. Considering the role of differentiation of CHS subtypes and the need to develop new treatment strategies, in this review, we introduced a multidisciplinary characterization of CHS from its pathology to therapies. We described the morphology of each subtype with the role of immunohistochemical markers in diagnostics of CHS. We also summarized the most frequently mutated genes and genome regions with altered pathways involved in the pathology of this tumor. Subsequently, we discussed imaging methods and the role of currently used therapies, including surgery and the limitations of chemo and radiotherapy. Finally, in this review, we presented novel targeted therapies, including those at ongoing clinical trials, which can be a potential future target in designing new therapeutics for patients with CHS.

## 1. Introduction

Chondrosarcoma (CHS) is the second most common sarcoma of bone, following osteosarcoma [1,2]. CHS accounts for approximately 27% of all bone cancer cases reported in the International Agency for Research on Cancer (IARC) database. However, substantial variability of proportions can be observed amongst countries, ranging from less than 10% of sarcoma cases in India and Saudi Arabia to over 45% in Finland, Slovenia, and the Netherlands. The overall age-standardized incidence rate (ASR) of CHS is 0.1–0.3 per 100,000 per year worldwide [2].

CHS primarily affects adults, unlike two other most common primary bone sarcomas (osteosarcoma and Ewing’s sarcoma). CHS is the most prevalent bone tumor in patients above 30 years of age, with over 70% of diagnosed cases present after 40 years. In children and young adults in the first two decades of life, CHS comprises only 3% of bone malignancies [3,4,5]. CHS incidence rate increases steadily with age [2,3]. Secondary CHS deriving from enchondromas generally affects younger patients than those with primary CHS [3].

CHS is slightly more common in males with a male-to-female ratio of 1.21:1 in England and 1.2–1.6:1 in the U.S. [5,6]. However, the incidence pattern of CHS has changed over time. The male predominance for CHS in the U.S. was more prominent before the 1990s. This ratio has diminished due to the increasing CHS incidence rate in females between 1996 and 2005 (from 0.16/100,000 to 0.27/100,000; estimated change: 3% per year), while incidence amongst men was generally invariable. Furthermore, both sexes’ upward trend was noted in England, Norway, and the Netherlands [5,7,8]. In the last case, the incidence rate increased markedly from 2.88 per million citizens between 1989–1996 to 8.78 between 2005–2013. Van Praag et al. [8] proposed the hypothesis of incrementing the number of imaging studies and progressive aging of society as possible explanations of this phenomenon. Moreover, increased exposure to exogenous estrogens (oral contraceptives and hormonal therapy) might be considered as a potential risk factor for CHS, although this supposition does not substantiate an increased incidence rate among men [2,6].

The data regarding ethnic predispositions for CHS are scarce. The study conducted using the New York State Cancer Registry showed a higher incidence of CHS amongst Caucasians than in African American residents with an age-adjusted rate (AAR) of 0.19 and 0.11 per 100,000, respectively [9]. Racial differences were likewise observed in the skull base CHS incidence [10]. Concerning the histological subtypes, significant disparities in incidence were noted in non-conventional CHS. The dedifferentiated subtype was more prevalent among Caucasians than in other populations, while the mesenchymal subtype was more common in the African American population [11].

CHSs constitute a heterogeneous group of locally aggressive and malignant bone tumors. It comprises neoplasms with distinct clinical and morphological features with a cartilaginous matrix production as a common trait [12]. Based on origin, CHS can be classified as primary if the tumor develops de novo or as secondary if it arises from pre-existing benign cartilaginous lesions—solitary or multiple osteochondromas and enchondromas. In approximately 80% of cases, secondary CHS occurs in osteochondromas, with slightly more frequent malignant transformation in solitary than multiple exostoses [1,13,14]. Reported cases also included synovial chondromatosis, and chondromyxoid fibroma rarely transforms towards secondary CHS. It is crucial to distinguish secondary CHS from dedifferentiated CHS, originating from the primary tumor, although it is associated with a poor prognosis. Secondary CHS affects younger patients than conventional primary CHS, with a mean age of 34 years at the diagnosis [1].

In most cases, due to indolent growth and rare metastases combined with the implementation of adequate treatment, CHS has an overall good prognosis [12]. The reported relative 5-year survival rate for CHS was 75.2% [4]. However, establishing an accurate prognosis for specific cases requires considering various factors. Taking into account the histological subtypes, the highest 5-year survival rates are observed in the periosteal subtype (80.3%, 68.1%) and conventional CHS (68.4%), followed by clear cell (62.3%), myxoid (64.1%, 49.8%), and mesenchymal (49.2%, 37.6%) subtype [4,11]. The lowest 5-year survival rates (11.3%, 17%, 24%) were noted in dedifferentiated CHS [7,11,15]. Regardless of the aforementioned significance in establishing the prognosis, grading is a subject of interobserver variability [16]. Thorkildsen et al. [17] proposed a novel system for risk stratification without the use of histological grade. The components of this system include the size of soft tissue component (< or ≥1 cm), axial or appendicular location, and presence of primary metastatic disease.

In the light of the heterogeneity of CHSs with the difference in occurring subtypes and grades, correlating with various prognostic factors, these tumors determinate difficulties in diagnostics and treatment. This review aims to provide an overview of the characteristics of all subtypes of CHS, including helpful diagnostic factors. In addition, this review outlines current trends in the treatment of these sarcomas along with potential therapies that have recently come to light and may in the future become the breakthrough in CHS treatment.

## 2. Morphology and Differentiation of Chondrosarcoma

CHS constitutes a various group of malignant bone tumors and encompasses multiple histological subtypes. Overall, conventional CHS is the most common subtype. It accounts for 85% of CHS, followed by dedifferentiated (10%), mesenchymal and clear cell CHS, these two representing less than 2% of all CHS cases [18,19]. Conventional CHS can develop de novo or in a pre-existing enchondroma (in Ollier disease or Maffucci syndrome; secondary central tumors) and osteochondroma (secondary peripheral tumors) [20,21]. CHS is classified into three grades based on cellular atypia, mitotic figures, and cellularity. The histological grading was based on the system proposed by Evans et al. [22]. CHS grade 1 and 2 are represented by 85% of all CHSs, 15% of cases are grade 3, and the dedifferentiated CHS [23]. According to the latest WHO recommendations, CHS should be classified into central or secondary peripheral atypical cartilaginous tumor/CHS (grade 1), central or secondary peripheral CHS grade 2 and 3 [20].

Secondary CHS may be associated with a genetic disorder called multiple hereditary exostoses (HME), characterized by the development of multiple osteochondromas affecting primarily metaphysis of the long bones. The most severe complication of this condition is malignant transformation into CHS, which ranges from 0.88% to 25.1% (on average 3.9%). In 75% of cases, transformation occurs in patients between 20 and 40 years. It involves principally appendicular skeleton (87.2% of cases) with the pelvis as the most frequent localization of secondary tumor (47.9%), followed by the scapula (12.3%) and proximal femur (8.3%). The spine and ribs accounted collectively for 12% of CHS [24]. Furthermore, the development of multiple enchondromas in nonhereditary disorders such as Ollier disease and Maffucci syndrome might also be a basis for secondary CHS with the transformation incidence rate of 5–50%. Verdegaal et al. [25] indicated that the occurrence of enchondromas in the pelvis is associated with a 3.8 times higher risk for developing malignancy in the skeleton. Moreover, patients with enchondromas present in the long bones or axial skeleton have a higher risk of transformation than those with lesions confined only to small bones of hands or feet [25]. The most common locations of secondary CHS in the diseases mentioned above encompass femur, humerus, tibia, pelvis, phalanges, and skull [25,26].

**Figure 1:**
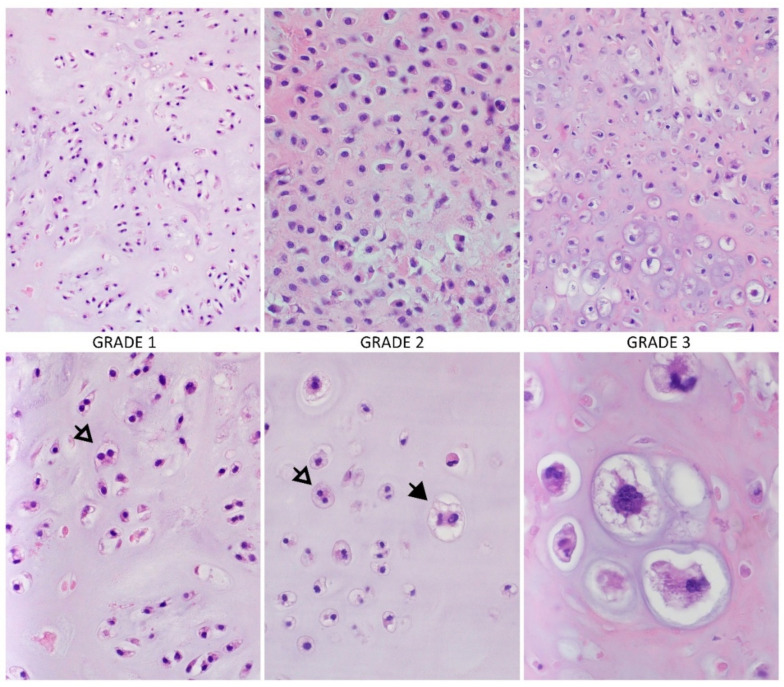
Conventional chondrosarcoma and histological grades. The higher-grade tumors are more hypercellular, with increasing mitotic activity, cytological and nuclear atypia, a matrix is changing from hyaline to more mucoid or myxoid (white arrow: binucleation of chondrocytes, black arrow: large, highly atypical chondrocyte; hematoxylin and eosin staining (HE), magnification: upper row 100×, bottom row 200× and 600×). Photos by Anna Szumera-Ciećkiewicz.

Macroscopically CHS is a large tumor, usually greater than 4 cm in size, with translucent lobular, blue-grey, or white cut surface corresponding to the presence of hyaline cartilage [27]. Microscopically, atypical chondrocytes with an irregular shape, size, and condensed binuclear nuclei with the necrosis area are observed [28]. Due to the production of the blue-grey cartilage-matrix by CHS, irregularly shaped and sized lobules of cartilage, separated by fibrous bands, are often found [28]. Liquefaction of the chondroid matrix and myxoid changes in higher-grade CHS are also frequent [28]. Other commonly observed features are the calcification areas, with the granular matrix consistence arising as a result of bone destruction by chondroid tissue. In contrast, nonmineralized areas have a translucent appearance [27]. Calcified areas in CHS suggest the enchondroma origin, but the differentiation is possible based on the relationship of the presence of cartilage tissue to the surrounding trabecular bone [27]. Microscopical features important in distinguishing between enchondroma and central CHS can also be higher cellularity, irregular distribution of the cells, and presence of binuclear [20]. According to histological differentiation, conventional CHS has 3 grades [20,27] (Figure 1):

Grade 1 (low), atypical cartilaginous tumor—often closely resemble normal cartilage or a benign type of enchondroma [27]. The cellularity is low to moderate, and chondrocytes have small, dense, and binuclear nuclei, usually not enlarged [28]. The mitotic figures are not present [22]. The stroma is generally in the majority a cartilage tissue; myxoid areas are usually sparse or absent [27].

Grade 2 (intermediate)—the cellularity is increased, which results in a smaller amount of chondroid matrix; the stroma is more frequently myxoid [27]. The nuclei are of moderate size; however, the mitotic rate is low (<2/10 HPF, high power fields). Cell nuclei are enlarged, vesicular, or hyperchromatic. Binucleated and multinucleated chondrocytes are common [27].

Grade 3 (high)—the cellularity is the highest, chondroid matrix is very scant with dominant myxoid areas [27]. The chondrocytes are irregular and spindle and tend to aggregate [27,28]. The nuclei are often vesicular and spindle-shaped and maybe 5 to 10 times larger than average [27]. The mitotic figures are frequently observed similar to necrosis areas [22,27]. Moreover, higher-grade CHSs have more extensive areas of non-calcified tissue [27].

**Figure 2:**
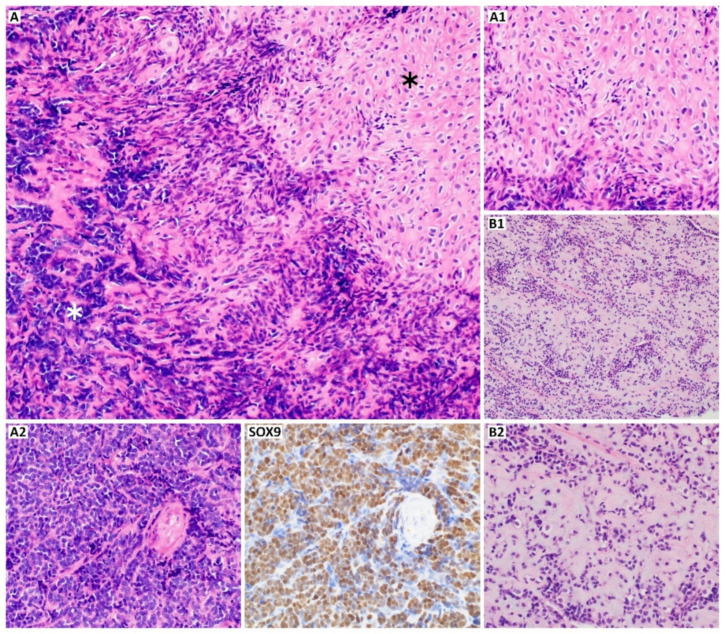
Mesenchymal chondrosarcoma ((**A**), HE, 200×). A mixture of round cells ((**A2**), white asterisk,; HE, 200×) with islands of well-differentiated hyaline cartilage ((**A1**), black asterisk; HE, 200×); the proportions of these two components may vary ((**B1**), HE, 100×; (**B2**)), HE, 200×); (**SOX9**) (SRY-Box Transcription Factor 9, 200×) is highly expressed supporting the differential diagnosis of mesenchymal chondrosarcoma. Photos by Anna Szumera-Ciećkiewicz.

**Figure 3:**
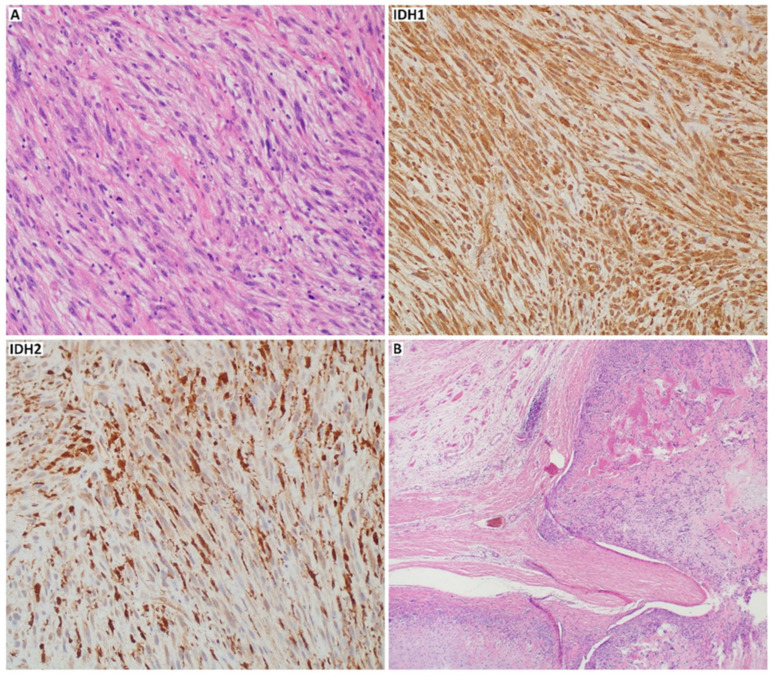
Dedifferentiated and periosteal chondrosarcoma. The transition to undifferentiated pleomorphic sarcoma ((**A**), HE, 200×), the case was positive with p.Arg132His mutation-specific isocitrate dehydrogenase 1 ((**IDH1**), 200×) antibody and negative for isocitrate dehydrogenase 2 ((**IDH2**), 200×); periosteal chondrosarcoma and cortical extension to soft-tissue ((**B**), HE, 40×). Photos by Anna Szumera-Ciećkiewicz.

High-grade, malignant tumors are also represented by mesenchymal and dedifferentiated CHS. The first shows a bimorphic histological pattern consisting of undifferentiated, small, round, uniform cells and well-differentiated hyaline cartilage areas [27,29]. Mesenchymal CHS stains positive for S100 and SRY-box transcription factor 9 (SOX9) (Figure 2), like conventional subtype, but it is also CD99 and NK2 homeobox 2 (NKX2.2) positive [30]. Furthermore, the co-expression of epithelial membrane antigen (EMA), mucin 1 (MUC1), desmin, myogenin, myoblast determination protein 1 (MyoD1) can be found. However, Friend leukemia virus integration 1 (FLI-1), smooth muscle actin (SMA), glial fibrillary acidic protein (GFAP), keratins, and integrase interactor 1 (INI1) are entirely negative for mesenchymal CHS [31]. Dedifferentiated CHS develops when a part of conventional low-grade CHS transits into aggressive, high-grade sarcoma (most commonly undifferentiated pleomorphic sarcoma, osteosarcoma, or other less frequent high-grade sarcomas, like angiosarcoma, leiomyosarcoma, and rhabdomyosarcoma) [32]. The non-cartilaginous component might be of different size and may correlate with the tumor malignancy [27,33]. Compared to the mesenchymal and conventional subtype, dedifferentiated CHS is negative for S100 in dedifferentiated components [34]. In about 20% of cases of dedifferentiated CHS, p.Arg132His mutation-specific isocitrate dehydrogenase 1 (IDH1) antibody may be positive (Figure 3). Both conventional and dedifferentiated components may express as well p53, mouse double minute 2 homolog (MDM2), programmed cell death receptor ligand 1 (PD-L1), and New York esophageal squamous cell carcinoma 1 (NY-ESO) marker [35,36,37].

Other histological subtypes, which are less frequent, are low-grade clear cell CHS and periosteal CHS. As the name suggests, the first one is characterized by a large number of transparent cells and clear, pale cytoplasm with the presence of glycogen and vacuoles [29]. The chondrocytes are visible as homogenous, large, and round cells with easily observed nucleoli inside the nucleus [27]. The clear cell CHS can be confused with osteoblastoma because of osseous metaplasia [27]. Periosteal CHS is marked by well-differentiated lobular and moderately cellular cartilage with calcification and endochondral ossification [33] (Figure 3B). The distinction between periosteal CHS and chondroma is based on invasion of the underlying cortex, observed in CHS, and tumor size [20].

Extraskeletal myxoid CHS is a malignant neoplasm of soft tissue, which has uncertain differentiation; it is characterized by multinodular architecture: abundant myxoid matrix and uniform cells arranged in cords and nuclear receptor subfamily 4 group A member 3 (*NR4A3*) gene rearrangement [38]. Regardless of the name, there is no proof of its cartilaginous origin.

The differentiation of CHS among other bone neoplasms is complex and based primarily on analysis of radiological images, clinical data, and evaluation of histological or molecular markers [39]. Although immunohistochemistry analysis can only support making a final diagnosis, some useful biomarkers may indicate CHS origin or correlate with histological grade (Table 1).

Firstly, it is worth mentioning S100 protein, expressed in many tumors, including several sarcomas [40,41]. This protein is related to a chondroid matrix and can be used as a marker for chondroid tissue origin. S100 protein expression was observed in many subtypes of CHS, like conventional CHS [34], clear cell CHS [42], mesenchymal CHS [43], and extraskeletal myxoid CHS [41]. On the other hand, S100 expression in enchondroma was not observed [44]. Other markers of chondroid differentiation are type II collagen or type X collagen [42], of which expression was described in conventional CHS [45]. Another marker correlated with osteochondral differentiation and cell proliferation is NEL-like protein 1 (NELL-1), expressed in benign and malignant bone tumors [46]. A subsequent marker, which can help differentiate low-grade CHS from enchondroma is periostin, enhancing tumorigenesis by increasing metastasis in several carcinomas [44]. The expression of this marker was observed only in CHS, in contrast to enchondroma [47].

Other essential markers are SOX4 (SRY-box transcription factor 4) and SOX9. The first was evaluated in cell proliferation, and apoptosis and its expression were correlated with histological grade [44]. The SOX9 is the central mediator of chondrogenesis, and it is expressed in all grades of conventional CHSs [48]. Its strong expression was also observed in the mesenchymal subtype [49]. Unfortunately, the SOX9 can also be expressed in osteosarcoma, synovial sarcoma, or other bone tumors, complicating the differential diagnosis [50,51,52].

To distinguish Ewing’s sarcoma from mesenchymal CHS, the transcription factor FLI-1 marker can be used [53]. In contrast to Ewing’s sarcoma, mesenchymal CHS lacks FLI-1. However, FLI-1 expression cannot help in small cell osteosarcoma and mesenchymal CHS differentiation [53]. On the other hand, the small cell osteosarcoma highly expresses special AT-rich sequence-binding protein 2 (SATB2), which regulates transcription and chromatin remodeling. SATB2 expression was two times lower in CHS (more frequent in high-grade) and rarely observed in Ewing’s sarcoma family tumors [54].

Moreover, markers that could distinguish subtypes of CHS were found as well. For example, Endo et al. [55] have shown that NY-ESO-1, a cancer-testis antigen, was expressed in conventional, dedifferentiated CHS and extraskeletal myxoid CHS. At the same time, its expression in clear cell and mesenchymal CHSs was not observed [55]. Another example of such markers is parathyroid hormone-related protein one receptor (PTHR1), expressed at a higher level in dedifferentiated areas of chondroid tissue than well-differentiated areas and other subtypes of CHS [56]. Additionally, catenin beta-1 (CTNB1) was higher expressed in small-cell areas of mesenchymal CHS and the dedifferentiated regions of dedifferentiated CHS, in comparison to a cartilaginous component of these subtypes and low-grade CHS, like clear cell CHS [56].

Many studies have also investigated the use of antibodies against proteins involved in the production of bone matrix. One of these proteins can be dentine matrix protein 1 (DMP-1) expressed by osteocytes, which can be used to distinguish between DMP-1 positive osteosarcoma and osteoblastoma from DMP-1 negative: CHSs, enchondromas, osteochondromas, melanoma, and some carcinomas, like for example, adenocarcinomas or renal cell carcinoma [35]. On the other hand, this marker cannot be used in differentiation between CHS and fibrosarcoma, Ewing’s sarcoma, or leiomyosarcoma [35].

Another point of interest can be cytoplasmic proteins such as ezrin. It is expressed in high-grade CHSs (mesenchymal, dedifferentiated), chondroblastoma, chondromyxoid fibromas, and chondroblastic osteosarcoma in contrast to conventional and clear cell CHS or osteosarcoma, which can help in differential diagnosis among these tumors [57]. The differentiation between chondroblastic osteosarcoma and conventional CHS was also found to be possible using galectin-1 (GAL-1), expressed by normal murine osteoblast and involved in cell growth differentiation, adhesion, migration, and apoptosis [58]. It has shown the strongest expression in chondroblastic osteosarcoma compared to conventional CHS [58]. GAL-1 was also expressed in the mesenchymal osteosarcoma component of dedifferentiated CHS [58].

In addition, an increased level of lactate dehydrogenase-A (LDH-A) was observed in CHS [59], which contributes to its treatment resistance [21]. In CHS, high expression of amphiregulin was also found, a ligand for epidermal growth factor receptor (EGFR), and correlates with histological grading in CHS [44]. Nevertheless, the utility of immunohistochemistry in the differential diagnosis of chondroid tumors remains limited.

**Table 1:** Comparison of markers in different subtypes of chondrosarcoma.

	S100	SOX9	Bcl-2	NY-ESO	P53	Mutated IDH	Others	References
**Conventional**	**+**	**+**	**+**	**+**	**+** (grade 2 and 3)	**+**	Brachyury, Col2a1, Cox-2, D2-40, Gal-1, MDM2, osteonectin, periostin, PTHrP, YKL-40	[34,41,44,45,48,55,58,60,61,62,63,64]
**Clear cell**	**+**	**+**	**+**	**−**	**+**	**−**	Col2a1, keratine, Runx2	[34,37,42,55,56]
**Mesenchymal**	**+**	**+**	**+**	**−**	**+**	**−**	CD99, desmin, EMA ezrin, MYF4, MYOD1, NKX2.2, vimentin	[30,31,34,55,56,57,60,63,65]
**Dedifferentiated**	**−**	**+** ^#^	**+**	**+**	**+** *	**+** **	CD44, Col1a1, Col2a1, cyclin D1, Ezrin, MDM2 *, PAI-1, PD-L1 ***, PTHR, Runx2	[34,36,37,45,55,56,57,60,63,66]

# SOX9 shows higher expression in the cartilaginous parts and lower expression in dedifferentiated compartments [45,56,67]. * p53 and MDM2 can be overexpressed in 59% and 16% of cases in dedifferentiated areas, respectively [37]. ** <20% of cases with IDH1 mutations can be identified by the p.Arg132His mutation-specific IDH1 antibody [60]. *** PD-L1 positivity was reported in 50% of cases [36]. Abbreviations: B-cell lymphoma 2 (Bcl-2), collagen type II alpha 1 chain (Col2a1), collagen type I alpha 1 chain (Col1a1), cyclooxygenase-1 (Cox-2), podoplanin (D2-40), equi merozoite antigen (EMA), galectin-1 (Gal-1), isocitrate dehydrogenase (IDH), mouse double minute 2 homolog (MDM2), myogenin (MYF4), myoblast determination protein 1 (MYOD1), NK2 homeobox 2 (NKX2.2), New York esophageal squamous cell carcinoma-1 (NY-ESO-1), plasminogen activator inhibitor 1 (PAI-1), programmed cell death receptor ligand 1 (PD-L1), parathyroid hormone-related protein (PTHrP), parathyroid hormone-related protein receptor (PTHR1), runt-related transcription factor 2 (Runx2), SRY-box transcription factor 9 (SOX9), chitinase 3-like 1 (YKL-40).

## 3. Genetics of Chondrosarcoma

There is still not much data about molecular abnormalities in CHS. Although no characteristic genomic changes are found, several mutations or chromosomal aberrations have been frequently observed in CHS, also among its subtypes.

**Figure 4:**
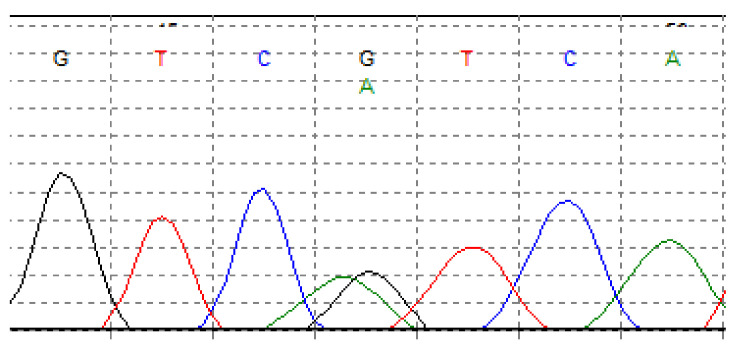
Somatic mutation in isocitrate dehydrogenase 1 (*IDH1*) in chondrosarcoma. G > A transition at nucleotide position 395, in codon 132, leading arginine to histidine substitution (p.Arg132His). Fluorogram by Andrzej Tysarowski.

**Figure 5:**
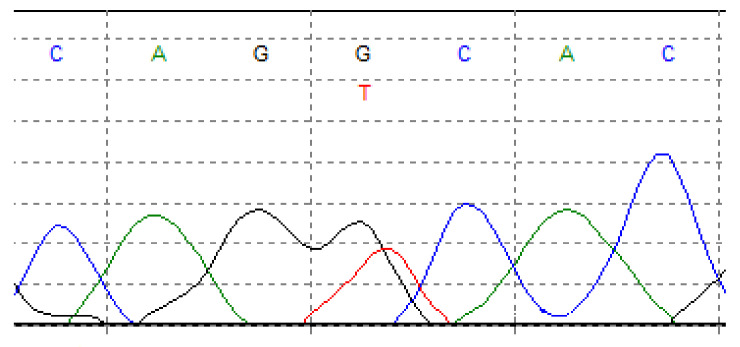
Somatic mutation in isocitrate dehydrogenase 2 (*IDH2*) in chondrosarcoma. G > T transition at nucleotide position 516, in codon 172, leading arginine to serine substitution (p.Arg172Ser). Fluorogram by Andrzej Tysarowski.

One of the most common point mutations occurs in *IDH1* and isocitrate dehydrogenase 2 (*IDH2*) genes. Isocitrate dehydrogenase is a metabolic enzyme that collateralizes the oxidative decarboxylation of isocitrate to alpha-ketoglutarate (a-KG). Mutations in *IDH1* and *IDH2* genes have been described in several malignancies, including gliomas [68], acute myeloid leukemia (AML) [69,70], and myelodysplastic disorders [71]. *IDH1*/*2* mutations are also documented in cartilaginous neoplasms, including approximately 50% of patients with CHS (65% of conventional CHSs and up to 57% of dedifferentiated CHSs) [60,72,73,74]. However, these mutations were not found in the clear cell [37] and mesenchymal CHS [60]. *IDH1*/*2* modifications included Arg132 *IDH1* (Figure 4), Arg172 *IDH2* (Figure 5), and Arg140 *IDH2* variants, with the majority of Arg132 *IDH1* variants [60,75]. Mutations in both these genes reduce the chances of survival in patients with CHS [28,75].

*IDH* mutations reduce a-KG activity, which leads to an elevated level of oncometabolite D-2-hydroxyglutarate (2-HG) [76]. The accumulation of 2-HG contributes to various changes in epigenetic pathways [76]. Li et al. [77] reported that 2-HG production, anchorage-independent growth, and cell migration were inhibited in cells without *IDH1* mutation. Furthermore, knocked-out mutant IDH1 CHS cell lines revealed the downregulation of several integrin genes [77]. Lugowska et al. [75], in their retrospective study, analyzed 80 patients diagnosed with CHS, and they have found the correlation between overall survival (OS) and the presence of *IDH1*/*2* mutations. These mutations were identified in 21% of grade 1 CHS patients and 44% of grade 3 CHS patients. The patients with activating *IDH1*/*2* mutations had a 5-year OS rate of 64% compared to 93% in those without mutations. In contrast to other studies, the frequency of *IDH1*/*2* gene mutations (34%), confirmed among patients analyzed in the same study, was lower than 50% [75]. The authors of other studies have noted higher rates of *IDH1*/*2* mutations—Amary et al. reported 56%, while Tallegas et al., found a prevalence of 65% [60,73]. However, all patients in the study of Lugowska et al. [75] suffered from peripheral CHSs, other studies reported *IDH1*/*2* mutations mostly in central CHS [60,73,75].

The presence of *IDH* mutations can also be used in differential diagnostic between dedifferentiated CHS and undifferentiated pleomorphic sarcoma of bone (UPS) [78] or high-grade CHS and chondroblastic osteosarcoma [60], where UPS and chondroblastic osteosarcoma lack *IDH1*/*2* mutations.

The second most frequent mutation in conventional (central and peripheral) and dedifferentiated CHS occurs in the *TP53* gene (20–50%) [21,66]. In multiple studies, the correlation between overexpression of the *TP53* gene or its alteration (loss of heterozygosity on chromosome 17p) and higher histologic grade of the tumor was observed [79,80,81]. This suggests the role of this gene in the tumor progression process [29].

Other frequently mutated genes in CHS, connected with the cell cycle control process, include *MDM2* and cyclin-dependent kinase 4 (*CDK4*), which inhibit p53 and are overexpressed in CHSs [79]. High expression of CDK4 and MDM2 is associated with amplification in the 12q13 gene region, which was often observed in central CHS and correlated with higher histological grade of this tumor [82]. The MDM2 overexpression was also observed in dedifferentiated CHS [37]; however, neither *CDK4* nor *MDM2* amplification in periosteal CHS was present [83]. The second crucial altered pathway involved in high-grade CHS is the retinoblastoma protein (pRB) pathway in which cyclin-dependent kinase inhibitor 2A (*CDKN2A*) gene, coding INK4 family member-p16 protein (p16INK4a) and p14ARF [82], plays the prominent role. In high-grade central CHS, the loss of expression of *CDKN2A*/p16/*INK4A*, caused by a deletion in the 9p21 region [79,82], is also a frequent event. Loss of *CDKN2A*/p16 was indicated in 25% of grade 2 CHS and even in 50% of grade 3 CHS, contrary to grade 1 CHS or enchondroma. This event was also detected in 62% of dedifferentiated CHS [84], around 30% of clear cell CHS, and 25% of mesenchymal CHS [37]. Deregulation of the pRB pathway by loss of p16 expression can also be involved in the pathogenesis of periosteal CHS [20].

Other gene alterations observed in CHS are amplification in the 8q24 region (the *c-MYC* oncogene) in about 33% of high-grade CHSs, and changes in 10 chromosome or 13q14 and 17p13 chromosomal regions, which occurs mainly in well-differentiated CHSs [28,85]. Amplification was also detected in the 11q22 region, affecting harboring a cluster of the matrix metalloproteinases (*MMP*) gene, highly expressed in CHS [86,87].

Moreover, the participation of some genes involved in cartilaginous tumors was also observed, such as inactivating mutations in exostosin-1/2 (*EXT1*/*EXT2*) genes. Those genes are involved in the proliferation of chondrocyte growth plate, mainly in secondary peripheral CHS [60,63,79]. Loss of heterozygosity and homozygous deletions of both *EXT1* and *EXT2* genes have been reported in peripheral CHS [87]. However, in this study, the expression of these genes was not significantly different between central and peripheral CHSs, compared to other findings [66,88]. *EXT* mutations are also found in osteochondroma but with much higher frequency than in CHS, suggesting the EXT-independent mechanisms of pathogenesis of secondary peripheral CHS [89]. It is also worth mentioning insertions, deletions, and rearrangements in the major cartilage collagen gene—collagen type II alpha 1 chain (*COL2A1*), identified in 37% of CHSs [66]. Mutations in the *COL2A1* gene were mainly found in central CHS; however, they also occurred in some dedifferentiated and clear cell CHSs [42,66].

Besides, many genes were observed to be overexpressed in CHS. For example, *AKT* (RAC-alpha serine/threonine-protein kinase) regulates multiple biological processes, including cell survival, proliferation, growth, and glycogen metabolism [29], was described. Associated with deregulation of the phosphatidylinositol 3-kinase (PI3K)-Akt pathway, phosphatase and tensin homolog deleted on the chromosome ten (*PTEN*) gene is downregulated in some CHSs, as a result of point mutations in this gene [90]. As a marker of poor survival and higher histological grade in CHS, a cyclooxygenase-2 (*COX2*) gene was also proposed (overexpressed in CHS) [29,82]. Overexpression was also observed in some oncogenes like Proto-oncogene tyrosine-protein kinase Src (*SRC*), which was noticed to be highly active in high-grade CHS [79]. Another example can be a nuclear expression of the ETS-related gene (*ERG*), which can be used as a potential marker to distinguish well-differentiated components of hyaline cartilage from dedifferentiated ones in high-grade CHS [91]. What is more, *ERG* was highly expressed in osteosarcoma, conventional, clear cell, and myxoid extraskeletal CHS, while in enchondroma, its expression was not observed [91].

In differentiation among sarcomas, gene fusions play a huge role; nevertheless, they are not well-known in CHS. One of the most common gene fusion recognized in mesenchymal CHS is between hairy/enhancer-of-split related with YRPW motif 1 (*HEY1*) and nuclear receptor coactivator 2 (*NCOA2*) genes, which is a deletion or translocation in the 8 chromosome region (q13;q21), involved in the transition of epithelial cells into its mesenchymal form [32,92,93]. Another recently discovered gene fusion in mesenchymal CHS is a translocation between chromosomes 1 and 5—t(1;5)(q42;q32)—concerning interferon regulatory factor 2-binding protein 2 (*IRFBP2*) and caudal type homeobox 1 (*CDX1*) genes [94]. Identified fusions with diagnostic potential, participating in several cellular processes such as cell divisions, differentiation, and apoptosis, were also found in extraskeletal myxoid CHS [32]. The following translocations in extraskeletal myxoid CHS were observed: t(9;22) (q31;q12) in EWS RNA binding protein 1 (*EWSR1*) and *NR4A3* genes, t(9;17)(q31;q12) in TATA-binding protein-associated factor 2N (*TAF15*) and *NR4A3* genes, t(9;3)(q31;q12) in trafficking from ER to Golgi regulator (*TFG*) and *NR4A3* genes, t(9;15)(q31;q21) in transcription factor 12 (*TCF12*) and *NR4A3* genes, t(9;11)(q31;q24) in heat shock protein family A (Hsp70) member 8 (*HSPA8*) and *NR4A3* genes, and t(9;16)(q31;q11) in FUS RNA binding protein (*FUS*/*TLS*) and *NR4A3* genes [32,95].

## 4. Imaging Methods in Chondrosarcoma

Early diagnosis is crucial for effective treatment and achievement of better prognosis in chondrosarcoma patients. The diagnosis of CHS can be challenging due to its diverse clinical manifestation with nonspecific symptoms, heterogeneous appearance in imaging studies, and variable biological behavior; therefore, a combination of these components must be considered to ascertain the proper treatment. One of the crucial factors is a differentiation between aggressive and more benign lesions. Particular traits of the lesions: larger size, localization in the pelvis and proximal parts of extremities, presence of multiple medullary lesions, and occurrence in patients over 50 years of age may indicate higher malignancy risk [96]. Common symptoms at presentation comprise pain, which may aggravate at night, palpable mass, limited range of motion of the affected joint, pathological fractures, swelling, and ailments associated with the mass effect and tumor location: dysuria, frequency, urinary incontinence, bowel obstruction, and numbness [13,14,96]. Most often diagnosed CHS are low- and intermediate-grade, with the most significant proportion of grade 2 lesions [7,18,97]. The tumor size varies between different histological subtypes and grades, ranging from 2 to 35 cm, with mean values of approximately 8–13 cm. The largest lesions are more prevalent in the dedifferentiated CHS, and their frequency increases from lower to higher-grade [7,11,98]. CHS primarily affects the appendicular skeleton. This location comprises more than 40% of cases [4,8,97], of which the most common sites are the long bones of the lower extremity, followed by the pelvis and the long bones of the upper extremity. Lesions arising in the ribs, sternum, and clavicles account for approximately 11–13% of cases [4,5,8], while tumors occurring in the skull are considered rare [99]. Additionally, the incidence of the CHS is not limited solely to the skeleton. The malignancy may also arise among other things in the soft tissue [97], skeletal muscles, brain, spinal cord [100], and larynx [101]. Several imaging modalities may detect, stage, characterize the tumors, and perform the image-guided biopsies [102]. One of the main challenges in chondrosarcoma imaging is to enable differential diagnosis between chondrosarcoma and benign tumors like chondroma [103]. The primary imaging modalities include radiography, computed tomography (CT), magnetic resonance imaging (MRI), scintigraphy, and positron emission tomography (PET).

The two techniques based on X-ray measurements, radiography, and CT, share the main features of CHSs. However, CT would be expected to be more reliable and sensitive, particularly in small tumors or the complex anatomical location of the tumor. CT is superior to radiography in the visualization of matrix mineralization [102]. CT is also typically used for image-guided biopsy [104]. Most frequently, CT/radiography of CHS would reveal a lesion with calcifications (“ring and arc” or “popcorn” pattern) and aggressive growth features; lytic lesions are also common [105]. However, the exact radiographic features would depend on the type of CHS and its grade. For example, intramedullary CHSs are typically characterized by mixed and sclerotic lesions with visible calcifications (mineralized chondroid matrix present in most cases). In the case of clear cell CHS, calcifications would be present only in 30% of cases. Dedifferentiated CHS is characterized by a heterogeneous pattern that would depend on the proportion of low- and high-grade areas in the lesion [102,106].

MRI is characterized by superior to other imaging techniques soft-tissue contrast. Therefore, it is well-suited to visualize soft tissue extension of CHS (e.g., present in the majority of diagnosed long bone conventional intramedullary CHSs [107]). This technique is also appropriate for the evaluation of bone marrow involvement. Marrow replacement by the lesion would be visible as low/intermediate signal intensity regions on T1-weighted images [27]. MRI also allows detecting peritumor edema, entrapped fat, and cortical damage [108]. It is frequently used for the evaluation of the lesion before surgery [102]. Its effectiveness in differentiation between other lesions might be enhanced by combination with radiographic methods [108].

CHS is typically visible on T1-weighted magnetic resonance (MR) images as a homogeneous signal that contrasts clearly with marrow signal; on T2-weighted images, the lesions are typically bright [109]. The calcified regions appear as foci of low-signal intensity appearing on the high-intensity chondroid components [27]. In post-contrast images (after gadolinium administration), CHSs show septal and peripheral enhancement (“ring and arc”) that is regarded not to be specific to these tumors but also appear in benign lesions [27,102]. In dynamic contrast-enhanced MRI (DCE MRI), CHSs are typically characterized by higher relative enhancement than muscle; the enhancement is also earlier and characterized by higher slope value [27]. Some studies suggest high specificity and sensitivity of DCE MRI in diagnosing CHS and differentiating between CHS and benign tumors. For example, in a study by De Coninck, et al. [110], 100% sensitivity and 63.3% specificity were reported. However, another study by Douis, et al. [111] failed to demonstrate the usefulness of DCE MRI in differentiation between the enchondromas and low-grade CHSs.

Several new MR-based approaches were proposed. It was suggested that magnetic resonance spectroscopy (MRS) techniques might differentiate between malignant bone tumors and benign lesions. Malignant bone tumors were shown to be characterized by higher choline signals than benign lesions [112]. MRS also showed elevated choline in CHS specimens [113]. However, the diagnostic value of localized ^1^H MRS needs to be evaluated in more extensive studies. One limitation of this approach could be the need to plan a relatively large volume of interest for the MRS study, i.e., in a range of 2 cm × 2 cm × 2 cm, to acquire a high-quality spectrum [112]. Another emerging approach is diffusion-weighted MRI [114], e.g., promising results were obtained in a skull base CHS, allowing to differentiate these tumors from chordoma [115]. However, another study failed in the differentiation of CHSs and benign bone lesions [116]. A promising approach could be the multiparametric MRI recently shown to effectively predict early recurrence (i.e., ≤one year) of pelvic CHS [117].

Several imaging techniques allowing detecting metabolic processes, based on the administration of radiotracers, are used to diagnose CHS. These techniques are sometimes referred to as functional imaging. The bone scintigraphy is based on the assessment of technetium 99m-methyl diphosphonate (Tc-99m MDP), thallium-201, or technetium-99m dimercaptosuccinic acid Tc-99m DMSA (V) [118]. The majority of CHSs are characterized by enhanced radionuclide uptake (compared to the anterior iliac crest) at bone scintigraphy [107,119]. A recent study demonstrated that elevated thallium-201 uptake was associated with almost eight times greater odds of grade 1 CHSs than enchondromas and elevated Tc-99m DMSA (V) uptake—nearly five times greater odds for CHS [120]. However, a limitation of bone scintigraphy is a subjective visual evaluation of the images and resulting inter-observer variability [119].

CHSs are regarded to be characterized by elevated uptake of another radiotracer, 18F fluoro-deoxyglucose (FDG), and to be detectable by FDG PET. The maximum standardized uptake value (SUVmax) seems to correlate with the grade of CHS, and the benign tumors would be rather characterized by lower SUVmax values [121]. Results published in a recent meta-analysis suggest that FDG PET/CT displays a high specificity and sensitivity in the diagnosis of CHS, superior to scintigraphy; it also seems beneficial for grading and monitoring the tumors [118,122]. However, it was also suggested that the value of SUVmax alone might not be sufficient to differentiate low-grade CHS from benign lesions [122].

The usefulness of quantitative single-photon emission computed tomography (SPECT) combined with CT for differentiating between enchondroma and grade I CHS was also recently evaluated [119]. The patients received metastable technetium-99m (Tc-99m) labeled diphosphonates, and the SUVmax values were significantly higher in the grade I CHSs than in enchondromas.

## 5. Surgical Treatment of Chondrosarcoma

Surgical excision remains the gold standard for the treatment of primary or recurrent CHS. The main goal of CHS surgery is resection of the tumor with the microscopically radical margins. According to Laitinen et al. [123], complete surgical excision reduces the risk of local recurrence (LR). However, it can be mutilating, functionally unacceptable, and not always technically or anatomically possible to obtain, even if we consider the recent advances in reconstructive surgery. It is well documented that having positive margins is related to a high chance for an LR and further distant metastases [123,124]. Unfortunately, regardless of overall improvement in the combined treatment of other types of bone sarcomas, we have not observed in the last 40 years any significant progress in CHS therapy. Thus the correct surgical approach is still the way to cure the majority of patients.

**Figure 6:**
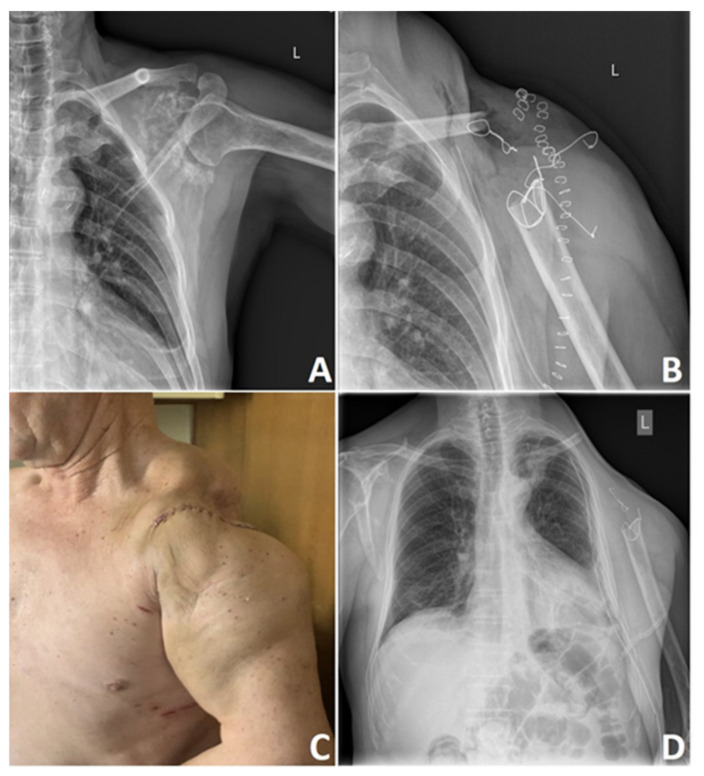
High-grade chondrosarcoma of the left scapula. Preoperative X-ray of the G3 scapular chondrosarcoma (**A**) and after en bloc upper interscapulothoracic humeral resection (**B**) This particular procedure known as Tikhoff-Linberg is a limb-sparing surgical option for tumors in and around the proximal humerus and shoulder girdle and an alternative treatment to forequarter amputation; however, is associated with poor cosmetic appearance (**C**) that can gradually change over the years depending on soft tissue contracture and scar tissue formation (**D**). Photos by Bartłomiej Szostakowski.

Poor vascularity of the tumor and low percentage of dividing cells significantly contribute to this tumor’s chemo and radiotherapy resistance [12,125]. There is a difference in treatment scenario between low-grade and high-grade CHS and axially and extremity located tumors. Aggressive high-grade tumors require aggressive surgical treatment, where adequate wide surgical margins remain the paramount factors of future results [126]. According to Fiorenza et al. [127], independent risk factors for LR are inadequate surgical margins and tumor size greater than 10 cm. Keeping in mind the low metastatic potential of the grade 1 CHS, it is currently apparent that wide surgical excision of small asymptomatic lesions that can be easily curetted or excised should not be a primary choice. In many cases, these tumors may be treated with contaminated margins to reduce operative morbidity and reduce patient survival. Of course, it is not a case for higher-grade CHSs where a wide excision is a necessary form of therapy [128] (Figure 6).

In the case of pelvic CHS, we often come across large tumors extending into the abdominopelvic cavity. Lack of significant anatomical barriers allows for a slow-growing tumor to expand into the cavity producing large extraskeletal masses that can abut essential structures such as bladder, rectum, or major vessels. In such cases, obtaining wide surgical margins may be problematic or impossible without mutilating surgery like hindquarter amputation [123,128]. As defined by Enneking classification, complete surgical excision is tumor removal with a sufficient cuff of normal tissue [129]. However, the definition of the adequate margin of soft tissue cover-age has never been specified. Enneking and Dunham, in their 1978 milestone article on resection and reconstruction within the innominate bone, have classified pelvic neoplasm resections into three main categories [129]:

- Type I: limited to the ilium.
- Type II: limited to the periacetabulum.
- Type III: limited to the pubis.

It is not always possible to adhere to these resection rules, especially in tumors with extensive soft tissue component that compromises neurovascular bundle [129]. However, according to Enneking classification, only tumors involving periacetabulum usually necessitate hindquarter amputation or limb-sparing reconstruction. Nevertheless, in terms of tumors with an extensive soft tissue component that grows in the vicinity of the critical anatomical structures or tumor that crosses compartments, we have to be flexible and modify our standards to achieve best results [129].

Although the bone margin is usually more comfortable to obtain, adequate soft tissue margin without compromising vital structures can sometimes pose a severe problem for the operating surgeon. Meticulous preoperative planning is key to better intraoperative performance and allows to achieve better long-term postoperative results. Critical preoperative evaluation of the most up-to-date imaging studies helps draw the proper surgical plan [118]. This entails the planning of the surgical biopsy. Except for standard radiographs in a minimum of two planes that can reveal areas of calcification or bony destruction, MRI is usually the best modality to assess the size, extent of the intraosseous and soft tissue involvement, and proximity of other anatomical structures [118].

Accurate preoperative grading of the tumor should be based on most recent imaging studies of various modalities and histopathology reports to avoid under- or overtreatment of the tumor. Distinguishing between enchondroma or low-grade CHS is usually very subtle and challenging in preoperative needle biopsy; therefore, both clinical and radiological findings support the final diagnosis [104]. Various studies confirm differences in grading based on preoperative needle biopsy results compared to final pathological reports in pelvic CHS. In some studies, biopsy concordance was less than 40% [104].

In recent years, we are observing the increased popularity of the computer navigated pelvic resections. Accuracy of the computer-guided surgery allows better oncological results, including increased free margin resections, decreased rate of LR, and better functional outcome [130,131,132]. According to Jeys et al. [133], computer navigated resections in primary bone sarcoma of the posterior ilium and sacrum, which usually pose a tremendous surgical difficulty, helped achieve tumor-free margins and improved disease-free survival (DFS) compared to non-navigated resections. As mentioned earlier, it is a generally accepted strategy that in selected patients with low-grade central CHS of long bones, curettage with adjuvant therapy remains a safe option [134].

**Figure 7:**
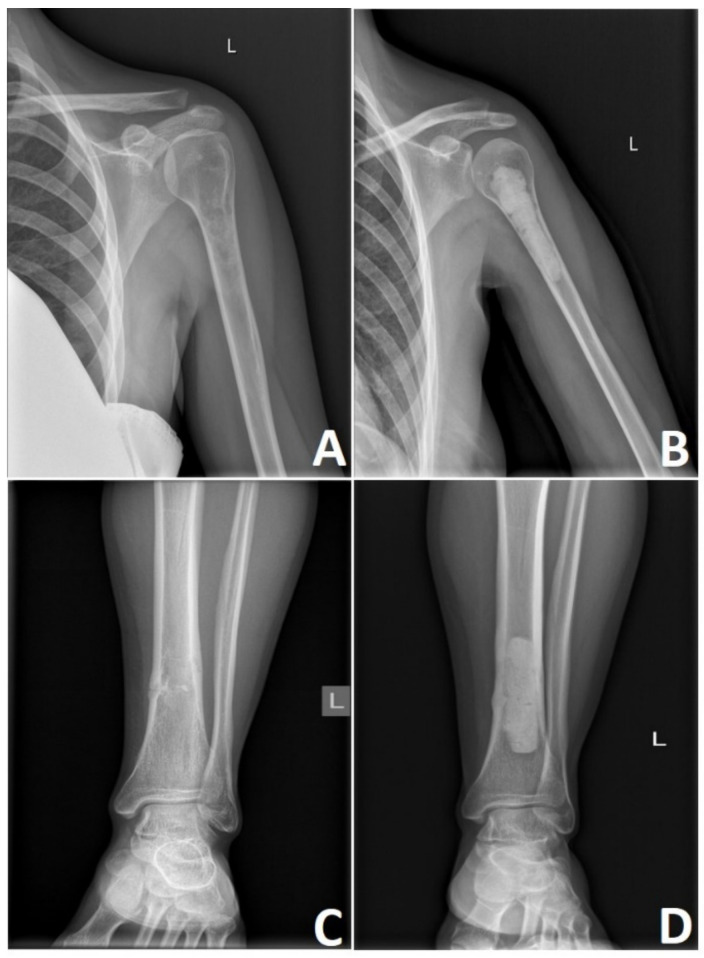
Low-grade chondrosarcoma. Rarely symptomatic lesions are discovered incidentally on X-rays after injury to the extremity (**A**,**C**). Meticulous curettage of G1 chondrosarcomas and polymethyl methacrylate cementoplasty remains the mainstay of treatment (**B**,**D**). Photos by Bartłomiej Szostakowski.

Specific adjuvants like polymethyl methacrylate (PMMA) bone cement, phenol, argon plasma, or cryotherapy applied at the time of intralesional resection can reduce the risk of LR, destroying microscopic disease left behind curettage. Meyer et al. [135] reported that PMMA bone cement could produce during polymerization relatively high temperature that can reach up to 107 degrees Celsius, causing thermal damage to the tumor cells and minimizing LR. Cementoplasty of the curetted tumor bed with PMMA continues to be a relatively safe and well-accepted solution for grade 1 CHS (Figure 7) [135].

**Figure 8:**
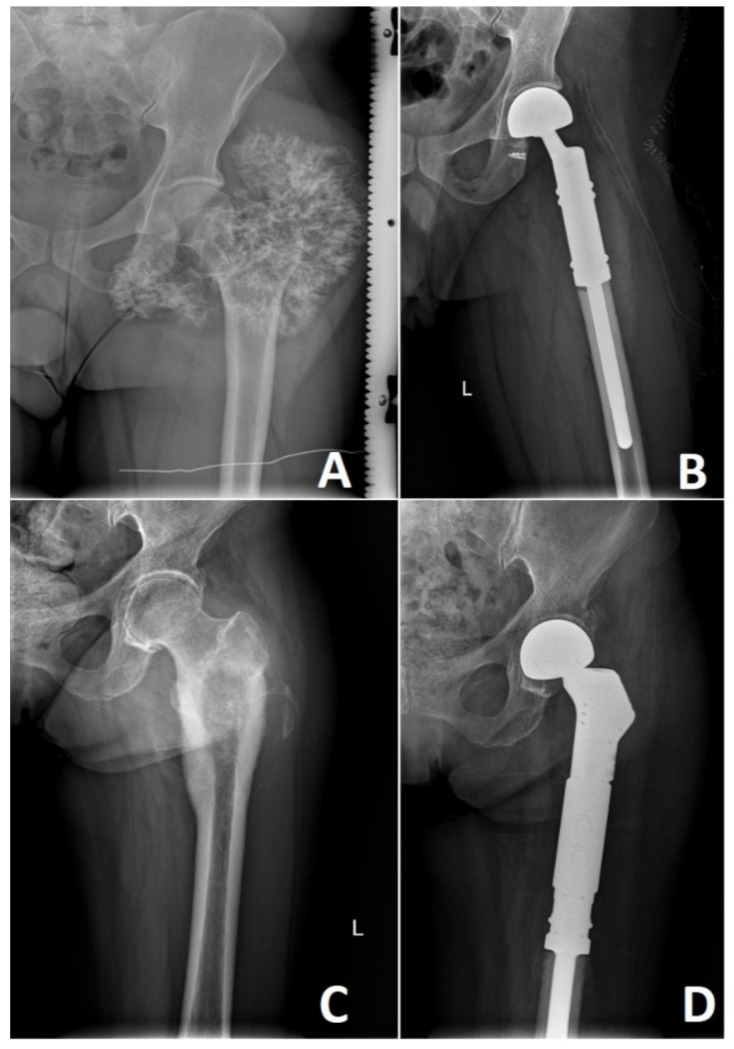
High grade locally advanced acral chondrosarcoma. Preoperative image of the locally advanced G2 (**A**) and G3 (**B**) chondrosarcoma of the left proximal femur. Reconstruction with modular endoprosthetic replacement (**C**,**D**). Photos by Bartłomiej Szostakowski.

High-grade CHS lesions of the long bones usually require resection followed by limb-sparing surgery. This involves reconstruction with a modular endoprosthetic replacement, custom-made prosthesis, or bone graft reconstruction (Figure 8). In rare occasions, the size or location of these tumors necessitates a more radical approach where amputation of the affected limb remains the treatment of choice. Bingold has reported one of the first resections of the long bone in CHS, followed by a custom-designed prosthetic replacement. After resection of the proximal femoral low-grade CHS, he reported prosthetic reconstruction that spans across 43 years with several revisions [136]. Nowadays, prosthetic replacements for low-grade CHS are advocated only in locally advanced lesions where curettage or marginal resection is not an option. Prosthetic reconstruction in high-grade CHS remains to be standard with multiple options available [136,137].

LR is a subsequent factor influencing the prognosis. LR develops in 14–26% of patients; nonetheless, this proportion varies depending on the CHS subtype, with the lowest rate in peripheral CHS (5–13%) and the highest in dedifferentiated CHS (42–55%), and the histologic grade with an incidence as follows: 12% in grade 1, 26% in grade 2, 37% in grade 3 [7,98,127]. This event occurs most often within approximately two years after the surgery [7,127]. The higher risk of LR is associated with the following tumor features: pelvic location, high grade, dedifferentiated subtype, and surgical margin of less than 1 mm in grade 1 CHS or of less than 4 mm in high-grade CHS [98]. This event correlates with a poorer prognosis—the 5-year survival rate for patients with LR was 55%, while in patients without LR, this rate was 87% [127]. Development of LR before one year after resection and age of 50 years or more are predictors of poor survival. Furthermore, the patients may develop subsequent LR to which predispositions constitute marginal or intralesional surgical margins for primary LR resection [138].

## 6. Radiotherapy in Chondrosarcoma

CHSs are considered radioresistant tumors. However, the mechanism of radioresistance has not been explained. Several explanations were proposed, including the ability to inhibit reactive oxygen species and high expression of anti-apoptotic genes [139,140]. Thus, radiotherapy in CHS is mainly used for metastatic disease as symptomatic treatment in selected cases after incomplete resection or unresectable tumors in anatomically challenging sites like the base of the skull or paraspinal area [141,142,143]. It has been shown that adjuvant radiotherapy after surgery for cranial CHSs can provide a substantial benefit in local control [144].

Due to the poor radiosensitivity, high total doses are recommended in definitive treatment (Table 2). Considering the dose-response relationship, the reasonable dose should exceed 60 Gy in 2 Gy fractions; however, even higher doses over 70 Gy are preferred [145]. In the selected cases, patients with unresectable tumors could benefit from debulking surgery followed by radiotherapy. Such a combined treatment facilitates radiotherapy by removing parts of the tumor adjacent to radiosensitive organs at risk, for example, optic chiasm. Local control after adjuvant radiotherapy is correlated with the residual tumor volume [146].

Altered fractionation regimens have not been validated in prospective clinical trials; however, they could be used in specialized centers concomitantly with modern radiotherapy techniques, allowing delivery of high dose with maximal sparing of surrounding healthy tissues [141].

**Figure 9:**
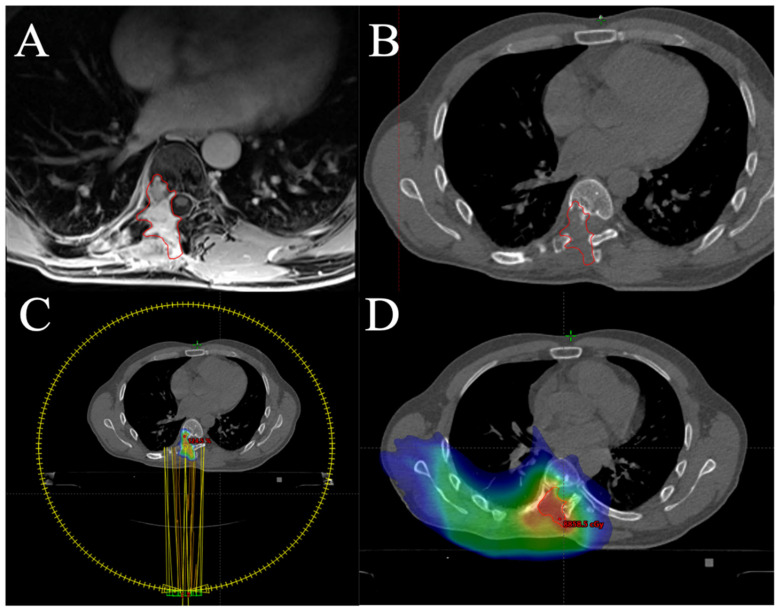
Stereotactic body radiotherapy (SBRT) for in-field recurrent high-grade mesenchymal chondrosarcoma (confirmed in next-generation sequencing sarcoma panel). In 2015, the patient underwent neoadjuvant chemotherapy followed by surgery, adjuvant radiochemotherapy (50.4 Gy in 1.8 Gy fractions with vincristine and dactinomycin combination chemotherapy), and chemotherapy according to EIAO regimen (etoposide, ifosfamide, vincristine, and dactinomycin). In 2019, he experienced local recurrence (LR) in the paraspinal area, which was resected in 2020. Five months later, the second LR was observed (**A**,**B**). The patients received SBRT (**C**) planned on fusion with magnetic resonance imaging and previous treatment plan (**D**). Volumetric arc therapy was used to deliver 30 Gy in five fractions prescribed on 80% isodose (**C**). Cord tolerance doses were compliant with the report published by Sahgal et al. [162]. Photos by Mateusz J. Spałek.

Particle therapy, namely proton therapy and carbon ion radiotherapy, uses unique physical properties called a Bragg peak [147]. It allows a deposition of most of the dose in precise volume followed by rapid distal fall-off. It enables delivery of the high dose to tumor volume while leaving normal tissues without harm in anatomically challenging areas. Moreover, carbon ions are characterized by high linear energy transfer. This enables a significant increase in relative biological effectiveness, being 2–3 times greater than in the case of photons. Carbon ions with high linear energy transfer cause hardly reparable complex DNA double-strand break that may overcome the radioresistance of tumor cells [147]. Both proton and carbon ion radiotherapy provide reasonable local control and patients’ survival rates with acceptable toxicity [148,149,150,151,152,153,154,155,156]. There is one ongoing prospective randomized phase III clinical trial that aims to compare the local efficacy of proton and carbon ion therapy in patients with low and intermediate-grade CHSs of the skull base (NCT01182753). Stereotactic body radiotherapy (SBRT) could also be a valuable treatment modality in selected patients with locally advanced or metastatic CHS [157,158,159]. Furthermore, SBRT may be carefully applied in patients with radiation-induced or in-field recurrent CHSs [159]; however, particular attention should be paid to the tolerance of vulnerable central nervous system structures and their ability to repair after prior radiotherapy [160,161,162]. An example of SBRT for in-field recurrent mesenchymal CHS is shown in Figure 9.

## 7. Chondrosarcoma Chemotherapy

Metastases are a negative risk factor associated with OS, disease-specific survival (DSS), and LR in CHS [7,127]. Fiorenza et al. [127] reported the development of metastases in 73% of patients with the LR, which resulted in a dismal prognosis—the individuals with both metastases and LR had a 5-year survival rate of 5% in comparison with 64% in patients with LR only. This event occurs on average in 15–32% of all cases and after approximately 2–4 years after implementing the therapy [7,127,163]. High tumor grade, dedifferentiated subtype, extracompartmental spread of disease, LR, and axial location in central CHS were associated with higher metastasis rates [7,127]. Furthermore, a group of patients with metastases at the time of diagnosis can be distinguished. It is characterized by poor overall 5-year and cancer-specific survival (CSS) rates of 28.4% and 31.2%, respectively. The negative predictors for these parameters are high tumor grade, size ≥10 cm, and lack of resection of the primary lesion [164]. The significance of primary tumor resection in improving the prognosis in this group was confirmed in patients younger than 65 years, with the conventional CHS and grade 2 malignancy. At two years for the patients after the surgery, the OS and CSS were 36.5% and 38%, while for those who did not undergo surgery, the values were 9.9% and 11.3%, respectively [165]. Regarding the location of metastasis, chondrosarcoma has a propensity for lungs, as it is the leading site involved by the malignancy. Other locations include bones, liver, skin, kidneys, and brain [166,167]. Chemotherapy appears to be of limited benefit as a treatment for CHS. To date, no effective systemic treatment has been identified in advanced or adjuvant phases for CHS. As a result, surgery remains the primary therapy. Nowadays, neither universal treatment guidelines nor chemotherapy regimens have been developed for advanced CHS patients. Chemotherapeutic options for CHS are not effective, but two histotypes appear to receive minimal benefits from chemotherapy. Namely, in some studies, mesenchymal and dedifferentiated CHSs have shown promising responses to chemotherapy [167].

Possible mechanisms underlying CHS chemoresistance include the expression of multidrug-resistance one gene P-glycoprotein [168], the high abundance of the cartilaginous matrix, the expression of anti-apoptotic proteins from the B-cell lymphoma 2 (Bcl-2) family, and the induction hypoxia-inducible factor 1α (HIF1α) by the high active kinase (AKT and SRC) [79].

In a large series of cases [167], the outcomes of 180 patients who had CHS and who were treated with chemotherapy have been retrospectively evaluated. Further, 73% of patients received an anthracycline-containing regimen, combination chemotherapy was administrated in 54.5% of cases, and the remainder had a single-agent therapy. Responses to chemotherapy were significantly different according to histological subtype and have been noted in mesenchymal (31%), dedifferentiated (20.5%), and conventional (11.5%) subtypes. These results found support in the subsequent analysis of 113 patients with mesenchymal CHS, where the reduced risk of recurrence and death were observed in patients who administered chemotherapy [169]. The prior studies also showed the role of chemotherapy in mesenchymal CHS therapy. Cesari et al. [170] provided evidence that DFS differs in patients with chemotherapy (76%) and without (17%) and D’Antonello et al. [171] showed the value for chemotherapy in the younger group of patients with localized disease.

Similarly, dedifferentiated CHS managed with a combination of surgery and chemotherapy may obtain a better clinical benefit than those treated without systemic therapy, as one retrospective evidence suggests [172]. Likewise, the outcome in a recent retrospective study described by Dhinsa et al. [173], including a subgroup of patients suffering from dedifferentiated CHS with osteosarcomatous dedifferentiation, compared favorably median survival in patients cured with chemotherapy (median of 17 months) to those without this treatment (median survival of 6 months).

Despite the data described above, no standard regimen has been established for managing these two histological types of CHS. Nevertheless, per current guidelines for treating bone cancers [141], dedifferentiated CHS should be treated based on the regimens for osteosarcoma. In contrast, mesenchymal CHS should be treated with Ewing’s sarcoma regimens. However, it is worth noting that the recommendations are categorized into 2B groups [141]. Of note, many CHS patients have a histological diagnosis of conventional CHS that is resistant to standard chemotherapy; thereby, this therapeutic option seems here to fail to demonstrate a clinically meaningful effect.

There are limited data on the efficacy of particular chemotherapeutic agents in this group of patients. Currently, cisplatin and doxorubicin are the primary recommended drugs for chemotherapy in CHS (CS); the recommendations were extrapolated from osteosarcoma treatment regimens [174]. Van Maldegem et al. [175] showed in the retrospective study the differences in outcome for progression-free survival (PFS) of the first systemic treatment line between patients diagnosed with conventional CHS treated with doxorubicin monotherapy and those treated with the combination of doxorubicin with cisplatin (2.5 vs. 3.6 months). Interestingly, the results were different in patients with dedifferentiated CHS—here, doxorubicin monotherapy seems to have a better PFS than a combination of doxorubicin with cisplatin (5.5 vs. 2.8 months). This is one of the most significant studies of systemic treatment outcomes in unresectable CHS, which has taken into account CHS subtypes and different treatment regimens. Prospective studies regarding the sensitivity of CHS to anticancer agents are scarce. To our knowledge, so far, only one prospective study [176] has been conducted to analyze the effect of chemotherapy (consisted of doxorubicin and cisplatin) for CHS. However, the study has included a small group of patients with two histopathological types of CHS—dedifferentiated and mesenchymal CHS; thus, the value of these two chemotherapeutic agents in the treatment was not determined.

Besides cisplatin and anthracycline, some CHS cell lines may display some sensitivity also to other anticancer agents. Ifosfamide and gemcitabine, drugs that have shown activity in other soft tissue or bone sarcomas, may have potential clinical activity in patients with primary resistance to anthracyclines [167].

The studies did not show the correlation between the number of chemotherapeutic agents used in a regimen and improved OS [167,175]. One of the studies has recently reviewed 865 patients with CHSs who received chemotherapy and an overall survival benefit being not observed [177]. The 5-year survival rate for stage III patients was 60.6% with chemotherapy and 58.6% without chemotherapy. For stage IV, significant difference between five-year OS for patients after chemotherapy and without chemotherapy was also not reported (28.2% vs. 31.2%) [177]. This underscores the urgent need for more effective therapeutic strategies for patients with CHS.

## 8. Targeted Therapies in Chondrosarcoma

### 8.1. Angiogenesis Inhibitors

One potentially effective target for the treatment of CHS is angiogenesis pathways. A positive correlation between CHS tumor grade and vascular endothelial growth factor (VEGF)-A expression has been reported, suggesting that antiangiogenic therapy could provide a therapeutic benefit in CHS [178]. Several encouraging clinical data have shown that antiangiogenic therapy could be a potentially helpful approach in CHS. Furumatsu et al. [179] assessed the angiogenic activities of a human CHS cell line (OUMS-27) in vivo and its effect on human umbilical vein endothelial cells (HUVECs) in vitro. They demonstrated that VEGF stimulates the proliferation and migration of endothelial cells, and VEGF targeted antibodies can restrict these endothelial responses by about 70%, suggesting the efficiency of antiangiogenic therapy targeting these molecules in CHS.

Morioka et al. [180] demonstrated the efficacy of combination antiangiogenic molecule therapy in CHS xenografts in mice. Furthermore, Levine et al. [181] also reported that antisense oligonucleotides against VEGF inhibit tumor cell proliferation in human cancer, including CHSs. Various antiangiogenic compounds in tyrosine kinase inhibitors and humanized monoclonal antibodies have been approved in several cancer treatments [174].

Orantinib (SU6668) is a multi-targeted receptor tyrosine kinase inhibitor which efficacy has been reported on CHSs in mice cells in vivo [182]. The anti-tumor activity of SU6668 has been attributed to its antiangiogenic potential, and a reduction in tumor vascularization demonstrated it.

In a retrospective search, Jones et al. [183] investigated the role of two antiangiogenic agents, pazopanib and ramucirumab, in advanced CHS. Ten patients diagnosed with CHS were identified and treated with antiangiogenic therapy. Pazopanib was prescribed at a fixed dose (800 mg per day) for eight patients, while the weight-adjusted dose of ramucirumab was administered every three weeks for two patients. Both drugs were reportedly well-tolerated; only one patient experienced grade 3 adverse events (AEs). No AEs lead to dose reductions, and no patient discontinued therapy due to toxicity. The most common were reported AEs to include hypertension and fatigue. None of the patients had a partial response; however, seven of them achieved prolonged disease stabilization for over half a year, with one patient treated with ramucirumab for 23 months. Median PFS was 22.6 months, and the median OS has not been demonstrated.

The monotherapy of pazopanib was rated in a prospective study [184]. Forty-seven patients with metastatic and surgically unresectable conventional CHS were enrolled and administered 800 mg of oral pazopanib once daily for 28-day cycles. Most of the patients (98%) reported AEs grade 2 or lower in severity, and 12 of them (26%) discontinued therapy due to toxicity. The most notable treatment-related AEs grade 3 was hypertension (12 patients [26%]), and elevated alanine aminotransferase levels (4 patients [9%]), and pulmonary emboli occurred in 2 patients (4%). The disease control rate (DCR) as the primary endpoint of this study was 43%. After four cycles of therapy (at week 16). The median OS was 17.6 months, and the median PFS was 7.9 months. Moreover, one patient had a partial response for 72 weeks.

Recently, regorafenib was evaluated as a novel therapy for patients with metastatic bone sarcomas, including CHS [185]. In a non-comparative, double-blind, placebo-controlled, phase 2 trial, this multitargeted tyrosine kinase inhibitor proved to be active in patients with progressive, metastatic osteosarcoma after the failure of conventional chemotherapy. Forty-three adult patients were recruited into the study. At eight weeks of treatment, the follow-up showed that 17 (65%) of 26 patients who received regorafenib were without disease progression. In contrast, progressive disease was reported in all patients in the placebo group. Median PFS was 16.4 weeks in the regorafenib group and 4.1 weeks in those randomly assigned to placebo. Twelve patients (46%) had AEs leading to transient withdrawal from the study. The following grade 3 or 4 AEs are the most common: hypertension (7 patients [24%]), hand-foot skin reaction (3 patients [10%] patients), fatigue (3 patients [10%]), hypophosphataemia (3 patients [10%]), and chest pain (3 patients [10%]). No treatment-related deaths were reported.

### 8.2. Cyclin-Dependent Kinase Inhibitors

Cyclin-dependent kinase inhibitors (CDKi’s) have shown efficacy in patients with breast cancer and are currently US Food and Drug Administration (FDA)-approved to treat this cancer. CDKs, beloning to the serine/threonine (Ser/Thr) protein kinases family, are regulatory enzymes that have critical roles in proliferation control and transcription [186,187]. They have been validated as oncogenic drivers in severity malignancies, and increased expression of them are frequently found in cancers [188,189]. CDK was also identified at enhanced levels in human CHS cell lines, and it was associated with malignant metastasis and undesirable prognosis of CHS patients [190]. These findings were presented in one clinical study [190], and palbociclib, the inhibitor of CDK4, was evaluated as a potential therapeutic for CHS patients. The authors indicated the activity of palbociclib in vitro, but future studies are warranted.

### 8.3. The Hedgehog Inhibitors

The hedgehog (Hh) pathway is a signaling cascade that plays a crucial role in many fundamental processes, including stem cell maintenance, cell differentiation, tissue polarity, and cell proliferation [191]. Misregulation of the Hh pathway has been found to cause neoplastic transformations, malignant tumors, and drug resistance of many cancers [192], also in CHS [193].

Indian hedgehog (IHH) and parathyroid hormone-related protein (PTHrP) are components of the negative feedback loop that control the development of plate chondrocytes [193]. Hh activates transcription—mediated by glioma-associated oncogene (GLI) downstream transcription factors—through binding to transmembrane receptors: patched protein 1 (PTCH1) receptor and smoothened (SMO) receptor [192,193]. This process leads to up-regulation of gene expression, including transmembrane protein PTCH1 and the transcription factor GLI-1.

Deregulation of this pathway during chondrocyte differentiation results in the secretion of PTHrP from chondrocytes and their proliferation. PTHrP inhibits proliferating chondrocytes from entering the prehypertrophic stage and reduces IHH production. Additionally, activated Hh signals implicate high expression of receptor PTCH1 and transcription factor GLI [193].

The role of the Hh pathway in CHS was also detected by Xiang et al. [194]. Focusing on primary cilia as part of the Hh signaling pathway, the authors evaluated the effect of Hh pathway inhibitor-4 (HPI-4) in regulating IHH-PTHrP signaling CHS cells in vitro. They suppressed their proliferation, invasion, and migration abilities [194].

Another study revealed the antitumor activity of saridegib (IPI-926), a potent oral Hh-inhibitor, in primary mice CHS xenografts [195]. Following IPI-926 treatment, ADAMTS like 1 (*ADAMTSL1*), one of the studied genes, was identified and was highly downregulated as a result of this therapy [195]. Despite that, the phase 2, double-blind, randomized, placebo-controlled study of IPI-926 in patients with CHS was stopped due to disappointing clinical data [196].

Further insight into the role of the Hh pathway in CHS was provided by the study of Sun et al. [197]. Curb the growth and survival of the CHS cells were observed due to knockdown of GLI-1 expression by siRNA and thus led to attenuation of the disturbed IHH signal pathway [197].

Unfortunately, the results of clinical trials with Hh inhibitors conducted so far have been discouraging. In a single-arm phase II trial, some activity of GDC-0449 (vismodegib), a small-molecule antagonist of the Hh signal pathway, was suggested to be presented in patients with grade 1 or 2 conventional CHS. In contrast, the primary endpoint has not been met [198]. The selection of patients more likely to benefit from this kind of targeted therapy with surrogate markers appears a feasible option for future studies in human CHS treatment [198].

The failures of clinical studies may be explained by occurring with PTCH or SMO mutation, which results in ligand-independent activation of the Hh pathway in CHS [199].

### 8.4. Histone Deacetylase Inhibitors

The histone deacetylase (HDAC) inhibitors have a significant role in gene expression, and aberrant activation of HDAC enzymes has been described in patients with various cancers [200,201]. Previous studies have shown that HDAC inhibitors impact various phenotypic changes, such as morphologic reversion of transformed cells, cell cycle arrest, apoptosis, and differentiation, which may contribute to neoplastic growth [202,203].

The FDA has so far approved the use of four different HDAC inhibitors to treat cancers. These include vorinostat (Zolinza) and romidepsin (Istodax) for the treatment of cutaneous T-cell lymphoma, and belinostat (Beleodaq) and panobinostat (Farydak) for the treatment of peripheral T-cell lymphoma and multiple myeloma [204,205,206,207].

Antitumor activity of an HDAC inhibitor was reported in preclinical studies on CHSs [208] and sarcoma cancer stem cells [209]. Based on these data, romidepsin was tested in phase II, in metastatic or unresectable soft tissue sarcomas, including extraskeletal CHS. Currently, the trial is completed, and the results are pending (NCT00112463).

Combination treatment with HDAC inhibitor—suberoylanilide hydroxamic acid (SAHA) and decitabine, and DNA methyltransferase inhibitor, were used on the three CHS cell lines: IDH wild type, IDH1 mutant, and IDH2 mutant cell line. The results were observed in increased levels of DNA damage marker, pro-apoptotic proteins, and apoptosis markers which led to decreased cell viability in all three tested cell lines [210]. Furthermore, in another study, class I HDAC inhibition sensitized CHS to glutaminolysis and Bcl-2 family member inhibitors, suggesting the role of HDACs in defining the apoptotic and metabolic state threshold in CHS [211].

Moreover, now another combination therapy is evaluating in phase 2 clinical trial (NCT04340843) with belinostat (HDAC inhibitor) and guadecitabine, the longer-acting hypomethylating agent.

### 8.5. IDH Inhibitors

Inhibition of mutated IDH1 in CHS cells has an adverse prognostic impact on survival; thereby, these findings open new clinical possibilities in managing CHS patients and improving outcomes for these patients.

In a study by Lugowska et al. [75], the presence of the R140 IDH2 mutation was identified in 3 CHS samples, and all of them were high-grade tumors. To date, this mutation has not been reported in CHS patients. However, this R140 mutation status might be linked with CHS grade, considering that the specific IDH2–R140 inhibitor, AG-221 (Enasidenib), could have the potential to offer therapy to patients with this genetic feature.

Ivosidenib (AG-120) is a targeted mutant IDH inhibitor that the FDA has approved for specific cases of acute myeloid leukemia. It has been studied in a phase 1 trial [74]. Twenty-one patients with mutant IDH1 CHS were enrolled in this study. The drug was administered orally in monotherapy in continuous 28-day cycles. Various escalating doses of the drug were evaluated, and the 500 mg orally once daily was selected to be most efficacious. Notably, the therapy was safe with no dose-limiting toxicities. Adverse events in a CHS population mainly were grade 1 or 2, and they were consistent with those seen in other mutant IDH solid tumors in this study. Ivosidenib demonstrated clinical activity, with a median PFS of 5.6 months and a 6-month PFS rate of 39.5%. Eleven (52%) of 21 patients experienced stable disease. Moreover, the study suggests that ivosidenib may be more effective in conventional CHS as PFS rates were higher in patients without dedifferentiated histology [74].

### 8.6. Tyrosine Kinase Inhibitors

Tyrosine kinase inhibitors (TKIs) represent the broad class of targeted therapies with multiple inhibitors approved to treat a broad range of different malignancies, including soft tissue sarcoma [212].

The effectiveness of the therapy with sunitinib has been observed retrospectively by Stacchiotti et al. [213] in small case series in a subtype-specific patient group with extraskeletal myxoid CHS. Besides the promising potency of dasatinib in CHS in the preclinical setting, it failed to demonstrate the clinically meaningful antitumor effect in CHS and other soft tissue sarcoma subgroups enrolled in the study [214]. The lack of activity has also been reported in the phase II trial of imatinib in patients suffering from this disease [215].

### 8.7. mTOR Inhibitors

The mammalian target of rapamycin (mTOR) has a crucial role in cell proliferation, cell survival, adhesion-independent survival, and migration. Dysregulation of the mTOR signaling, as reflected by elevated phosphorylation of eukaryotic translation initiation factor 4E-binding protein 1 (4E-BP1) and ribosomal protein S6 kinase beta-1 (S6K1), has been reported in various cancers [216,217,218]. One study investigating synergistic anti-tumor activity in the combination of mTOR inhibitor—sirolimus and cyclophosphamide in CHS patients showed the clinical benefits with a disease control rate of 70% [219].

Everolimus is another mTOR inhibitor approved by the FDA to treat various cancers, including breast, renal carcinoma, and pancreatic neuroendocrine tumor. It was tested in vivo in CHS models as a single agent and in combination with doxorubicin. The study indicated that everolimus blocks tumor progression with no synergistic additive effect with doxorubicin [220]. It suggests that there is a place for an mTOR targeted therapy in the management of CHS.

### 8.8. Osteoclast Inhibitors

The presence of osteoclasts, the bone-resorbing cells, has been observed in the microenvironment of CHS. They induce pathogenetic processes responsible for the bone destruction behavior, and their activity contributes to the growth of CHS in bone [221]. Consistent with these findings, the authors of one study utilizing the Swarm rat CHS model demonstrated osteoclast-targeted drugs as a potentially helpful approach in CHS [221]. Thus, zoledronic acid, an inhibitor of osteoclasts, has been shown as an adjunct therapy in various tumors affecting the bones, including CHS [222,223,224].

### 8.9. Immunotherapy in Chondrosarcoma

Recently, cancer immunotherapies have received considerable attention due to their notable success in treating a variety of human cancers, such as metastatic melanoma, renal cell carcinoma, lung cancer, and breast cancer. The potential use of immunotherapy in other cancer types has received a considerable amount of interest, leading to the rapid expansion of research assessing immune checkpoint blockade potential and CHS management [19]. Few data are available for this newer therapy in CHS, but most of the studies have included mesenchymal and dedifferentiated CHS patients. These types of CHS have been noted to be different from conventional CHS in response to chemotherapy, and they represent less than 15% of all CHSs. Nevertheless, Kostine et al. [36], in their research, revealed PD-L1 expression in a significant proportion exclusively in the dedifferentiated subtype. The presence of this biomarker was identified in 41% (9 of the 22) of the tested cells, and it was confirmed in the analyses of whole tissue sections in which PD-L1 expression was detected in 52% (11 of the 21) of cases. The PD-L1 positivity was correlated with high T-cells and macrophage infiltration, as well as high HLA I expression [36]. These findings were consistent with the outcomes from the phase 2 trial with pembrolizumab in patients with advanced soft tissue and bone sarcoma. The number of patients of CHS (5 of the 86) in this study was not prespecified, although one of two confirmed partial responses was observed in one patient (20% of patients) with CHS [225]. Similar results were seen in one retrospective study in which one of four patients (25%) with dedifferentiated CHS responded to nivolumab [226]. To date, immune checkpoint inhibitor therapy in conventional CHS has been presented only in one published report [227], which shows the favorable response to nivolumab in one patient with advanced conventional CHS. These findings collectively highlight that further evaluation of immune checkpoint blockade in CHS is highly needed (Table 3).

## 9. Conclusions

CHSs are a very heterogeneous group of bone tumors. Although CHSs occur rarely, they constitute a serious problem due to their malignancy and difficulties in diagnosing and treatment. Due to the rare occurrence of these tumors, detailed data about their pathology and therapeutic management are still limited. The assessment of the prognostic factors reported in the literature is inconsistent due to the heterogeneity of the cohorts between studies and the fact that research is often limited only to a single institutional experience [17,97]. One of the nationwide studies conducted by Giuffrida et al. [97] utilizing the Surveillance, Epidemiology and End Results (SEER) database with 2890 CHS cases indicated only two independent predictors of survival—histological grade and surgical stage. Grade 1 and 2 tumors have significantly higher DSS and 10-years survival rates than grade 3 tumors [97,98,127]. Due to a study by Nota et al. [15], histological grades are very various and correlated with survival as follows: 5-year survival for grade 1 CHS ranged from 82% to 99%, grade 2–63% to 92%, grade 3–0% to 77%, as the series of 1114 patients reported. Moreover, a better prognosis is characterized by the lower surgical stage in the American Joint Committee on Cancer (AJCC) staging system. Patients with localized disease (M0) had a higher thirty-year survival rate than those with regional (M1) and metastatic (M2) disease (43%, 22.30%, and <10%, respectively) [97]. Another research with a cohort comprising all CHS subtypes demonstrated that DSS is dependent on the histological subtype, location of the tumor (central axial lesions had a worse prognosis than central peripheral), age, metastasis at diagnosis, and presence of a soft tissue component [7]. Gender was associated with survival only in univariate analysis [97]. The prognostic factors of the particular subtypes were also proposed. For conventional CHS, negative prognostic factors encompass increasing age, tumor size greater than 8 cm, location in pelvis, sacrum, coccyx, and spine (compared to extremities) [8]. The common factor for non-conventional CHS subtypes constitutes the presence of metastases. The high-grade status was significant only in clear cell and myxoid CHS [11]. Currently, more cases and clinical data are still needed to study the treatment of CHS and to define positive and negative factors affecting prognosis in these patients.

There is a huge need to provide new research on CHS’s pathology and develop new molecular targets, which can be helpful in improving present and often invasive treatment methods of this disease. The improved preselection in vitro experiments of new therapeutic options is needed, and preclinical testing should increase the specificity of information available for in vivo animal testing and, later, clinical trials [228]. More efficient, cost-effective, and ethically beneficial methods in CHS pre-clinical studies are needed. Until now, there are no large-scale clinical reports on the relationship between systemic treatment and prognosis in CHS patients [229].

In the future, targeted therapies based on inhibitors of proteins involved in altered molecular pathways and mutated genes (e.g., kinase inhibitors, HDAC inhibitors, hedgehog inhibitors, and mutated IDH inhibitors) may be a point of interest. Increasingly, there is also a need for personalized therapies, especially in many tumors. At the same time, only a few studies have analyzed sarcoma stem cells or sarcoma-associated stroma and its impact on drug response, and such investigations are needed in CHS. Finally, the immune-phenotype of CHS has not been fully characterized. Macrophages or NK cells may contribute to the immune surveillance in CHS, but have not been fully characterized [230]. Therefore, there has recently been growing interest in immunotherapy and immune checkpoint blockade, such as PD-1 inhibitors, which can also be beneficial in future CHS treatment. Moreover, it has been suggested that the combination of nano- and immuno-therapeutics could become a breakthrough therapy in unresectable CHS. The first example of novel treatment may be nivolumab combined with nanoparticle albumin-bound rapamycin—a combination that has shown first promising clinical outcomes [231]. Other novel drug candidates in the field of CHS include INBRX-109—tetravalent single domain antibody (sdAb) that agonizes death receptor 5 (DR5) to induce CHS cells selective programmed cell death, as presented in 2020 CTOS Virtual Annual Meeting. After the successful phase 1 trial, a potential registration-enabling phase 2 study of INBRX-109 has been discussed with the FDA and is expected as a randomized, blinded, placebo-controlled study for patients with unresectable or metastatic conventional CHS with PFS as the primary endpoint. In the phase 1 trial, disease control was observed in 11 of 12 patients (92%) and 8 of 12 patients (67%) achieved overall response rate (ORR). In fact, Global Chondrosarcoma Pipeline Insight Report 2021 (Business Wire) indicates vismodegib, Aldox, INBRX-109, AB 218, and Ifosfamide as current key products for CHS and Roche, ImmunityBio, Inhibrx, AnHeart Therapeutics and Atlanthera as key players. Drug repurposing may also become as a source of innovative therapies in CHS as in the first screen of 255 drugs, 19 have potential activity [232].

## Figures and Tables

**Table 2:** Recommended doses in 1.8–2 Gy fractions in definitive radiotherapy for chondrosarcoma. Based on [141,142,143].

Recommendations	Sequence	Microscopically Positive Margins	Macroscopically Positive Margins	Unresectable Tumors
National Comprehensive Cancer Network	perioperative	preoperative: 19.8–50.4 Gy boost: up to 70 Gy	preoperative: 19.8–50.4 Gy boost: up to 72–78 Gy	not applicable
	postoperative	70 Gy	>70 Gy	>70 Gy
Scandinavian Sarcoma Group	postoperative	56–62 Gy	64–70 Gy	≥70 Gy
Spanish Sarcoma Group	postoperative	>60 Gy	>60 Gy	>60 Gy 40–70 Gy *

* Described as palliative radiotherapy.

**Table 3:** Ongoing clinical trials for chondrosarcoma. Twenty-three (2 with radiation therapy; 21 with novels) clinical trials for chondrosarcoma were found using the following keywords: chondrosarcoma, bone sarcomas, solid tumors (https://clinicaltrials.gov and https://www.clinicaltrialsregister.eu/, accessed on 3 April 2021).

Clinical Trial	Agent/ Interventions	Phase	Study Population	Status
NCT04278781	AG-120	Phase 2	IDH1 mutant chondrosarcoma	Recruiting
NCT02389244	Regorafenib	Phase 2	Metastatic bone sarcoma, chondrosarcoma	Recruiting
NCT04040205	Abemaciclib	Phase 2	Advanced bone sarcoma, including chondrosarcoma	Recruiting
NCT03173976	Zoledronic acid	Phase 1b	Resectable chondrosarcoma	Recruiting
NCT04340843	Combination belinostat and guadecitabine	Phase 2	Conventional Chondrosarcoma	Not yet recruiting
NCT03277924	Nivolumab plus Sunitinib	Phase 1/2	Advanced bone sarcomas	Recruiting
NCT03190174	Combination nivolumab and nab-rapamycin (ABI-009)	Phase 1/2	Advanced malignancies, including sarcomas with deficient mismatch repair	Active, not recruiting
NCT03474640	Toripalimab	Phase 1	Advanced malignancies including chondrosarcoma	Recruiting
NCT04690725	TQB3525	Phase 1 Phase 2	Advanced Bone Sarcomas	Active, not recruiting
NCT04762602	HMPL-306	Phase 1	Isocitrate Dehydrogenase Gene Mutation	Not recruiting
NCT03670069	Itacitinib	Phase 1	Metastatic Chondrosarcoma Sarcoma Tumor Immune Microenvironment	Recruiting
NCT03449108	Aldesleukin Autologous Tumor-Infiltrating Lymphocytes LN-145 Autologous Tumor-Infiltrating Lymphocytes LN-145-S1 Cyclophosphamide Fludarabine	Phase 2	Bone Sarcoma Dedifferentiated Chondrosarcoma Giant Cell Tumor of Bone Malignancy in Giant Cell Tumor of Bone Malignant Solid Neoplasm	Recruiting
NCT03684811	Drug: FT-2102 Drug: Azacitidine Biological: Nivolumab Drug: Gemcitabine and Cisplatin	Phase 1 Phase 2	Cohort 1a and 1b: Glioma Cohort 1a and 1b: Glioblastoma Multiforme Cohort 2a and 2b: Hepatobiliary Tumors (Hepatocellular Carcinoma, Bile Duct Carcinoma, Intrahepatic Cholangiocarcinoma, Other Hepatobiliary Carcinomas) Cohort 3a and 3b: Chondrosarcoma Cohort 4a and 4b: Intrahepatic Cholangiocarcinoma Cohort 5a: Other Solid Tumors With IDH1 Mutations	Recruiting
NCT04521686	LY3410738	Phase 1	Cholangiocarcinoma Chondrosarcoma Any Solid Tumor	Recruiting
NCT04305548	Trabectedin	Phase 2	Advanced Rearranged Mesenchymal Chondrosarcoma	Not yet recruiting
NCT04458922	Atezolizumab	Phase 2	Chondrosarcoma NCI Grade 2 Chondrosarcoma NCI Grade 3 Clear Cell Sarcoma of Soft Tissue Dedifferentiated Chondrosarcoma Primary Central Chondrosarcoma	Recruiting
NCT01267955	Vismodegib	Phase 2	Clear Cell Chondrosarcoma Dedifferentiated Chondrosarcoma Locally Advanced Chondrosarcoma Mesenchymal Chondrosarcoma Metastatic Chondrosarcoma Primary Central Chondrosarcoma Unresectable Primary Central Chondrosarcoma	Active, not recruiting
NCT02821507	sirolimus and cyclophosphamide	Phase 2	Conventional Chondrosarcoma Myxoid Liposarcoma Mesenchymal Chondrosarcoma Dedifferentiated Chondrosarcoma	Recruiting
NCT02066285	Pazopanib	Phase 2	Solitary Fibrous Tumor Extraskeletal Myxoid Chondrosarcoma	Active, not recruiting
2010-019817-20	GDC-0449	Phase 2	Advanced chondrosarcomas	Ongoing
NCT04260113	Apatinib Mesylate	Not Applicable	Unresectable Advanced Chondrosarcoma	Active, not recruiting
NCT02073994	AG-120	Phase 1	Cholangiocarcinoma Chondrosarcoma Glioma Other Advanced Solid Tumors	Active, not recruiting
NCT02048371	Regorafenib	Phase 2	Liposarcoma Osteogenic Sarcoma Ewing’s/Ewing-like Sarcoma Rhabdomyosarcoma Mesenchymal Chondrosarcoma	Recruiting
NCT01182753	Radiation: carbon ion therapy Radiation: proton therapy	Phase 3	Chondrosarcoma	Recruiting
NCT02838602	Radiation: Carbon ions therapy Radiation: Advanced external radiotherapy by Xrays or protons	Not Applicable	Malignant Tumors as Chordoma, Adenoid Cystic Carcinoma, and Sarcoma	Recruiting
